# Anterior release is not needed to restore kyphosis in moderate AIS with hypokyphosis

**DOI:** 10.1007/s43390-025-01119-7

**Published:** 2025-06-03

**Authors:** Craig R. Louer, Jacquelyn S. Pennings, Maty Petcharaporn, Arun R. Hariharan, John S. Vorhies, Michael P. Kelly, Suken A. Shah, Peter O. Newton, Burt Yaszay, Harms Study Group

**Affiliations:** 1 Department of Orthopedic Surgery, Vanderbilt University Medical Center, Nashville, TN USA; 2 Department of Biostatistics, Vanderbilt University Medical Center, Nashville, TN USA; 3 Center for Musculoskeletal Research, Vanderbilt University Medical Center, Nashville, TN USA; 4 Setting Scoliosis Straight Foundation, San Diego, CA USA; 5 Paley Orthopedic and Spine Institute, West Palm Beach, FL USA; 6 Department of Orthopedics, Stanford University, Palo Alto, CA USA; 7 Rady Children’s Hospital, San Diego, CA USA; 8 Nemours Children’s Health, Wilmington, DE USA; 9 Division of Orthopaedic Surgery, University of Washington School of Medicine, Seattle, WA USA

**Keywords:** Adolescent idiopathic scoliosis, Anterior release, Discectomy, Kyphosis, Thoracic lordosis, Posterior spine fusion

## Abstract

**Purpose:**

The purpose of this study is to evaluate if AR offers improved 3D kyphosis restoration during PSF for hypokyphosis in moderate AIS (< 70° coronal cobb), where the decision for AR is likely driven by sagittal concerns.

**Methods:**

A multicenter pediatric spine registry was queried for hypokyphotic (< 10°) Lenke 1–4 AIS patients aged < 20 years with > 2-year surgical follow-up. Coronal curves were limited to < 70°. A linear mixed model was created to predict 2-year 3D kyphosis by treatment and pre-op 3D kyphosis, while controlling for age, sex, thoracic coronal deformity and flexibility, osteotomy use, implant characteristics, surgery recency, and surgeon.

**Results:**

1384 patients were included with 53 (3.8%) undergoing PSF + AR. Mean preop 3D kyphosis was similar between PSF and PSF + AR groups (− 3.7° vs. − 0.5°; *p* = 0.08). PSF-AR had similar 2-year 3D kyphosis (23.0° [95% CI 20.5–25.4°] vs. 23.3° [22.9–23.6°]) and correction (26.7° [23.3–29.9°] vs. 23.7° [23.3–24.2°]) compared to PSF. When controlling for covariates, the models demonstrated no difference between approach (*p* = 0.058) or interaction of approach and preop 3D kyphosis (p = 0.31). Post-hoc power analysis showed an adequate sample size to detect a difference of 5° between approaches. PSF + AR had longer surgical times (324 vs. 266 min, p < 0.001) though no significant increase in overall complications (17% vs. 12.4%) compared to PSF alone.

**Conclusion:**

In AIS patients with coronal curve < 70° and 3D hypokyphosis of 10 to − 40°, treatment with PSF + AR did not improve 2-year sagittal correction more than PSF alone. Surgeon identity and surgery recency influenced post-operative kyphosis more than any other patient or surgical factor.

## Introduction

Adolescent Idiopathic Scoliosis (AIS) is increasingly recognized as a three-dimensional (3D) deformity consisting of characteristic coronal angulation in addition to axial rotation and loss of thoracic kyphosis in the sagittal plane [1]. Severe thoracic hypokyphosis can lead to pulmonary restriction [2, 3] and undesirable compensatory loss of lordosis in the lumbar or cervical spine, which has been linked to degenerative changes in these regions. [4–6] Restoration or preservation of thoracic kyphosis, therefore, remains a focus of 3D AIS correction surgery [7].

Bradford and colleagues pioneered the surgical technique of anterior spine release (AR) combined with posterior spine fusion (PSF) for AIS with severe thoracic lordosis in 1983 [8]. Though effective in this series, AR is an additional surgical approach and procedure with increased surgical effort and time, as well as additional risks [9]. With surgical advances in segmental fixation, thoracic pedicle screws, increasing rod diameter, stronger materials, and posterior release techniques, the efficacy and necessity of anterior release has been debated [9–11].

These prior studies evaluating the efficacy of AR have focused primarily on large (≥ 70°), stiff coronal deformities where the coronal plane drives the decision for PSF with AR. When the sagittal plane was considered, it was considered in two dimensions, which can underestimate 3D hypokyphosis when coronal and axial planes remain uncorrected. With increasing recognition of AIS hypokyphosis and the growing importance of kyphosis restoration, the role of AR for these deformities is understudied.

The purpose of this study is to evaluate if AR offers improved 3D kyphosis restoration during PSF for hypokyphosis in moderate AIS (< 70° coronal cobb), where the decision for AR is likely driven by sagittal concerns. We hypothesize that PSF + AR and PSF alone result in similar 2-year postoperative 3D kyphosis for mild deformities, but that more severe lordotic deformities will benefit from improved correction with the addition of AR. These results can better define the severity of hypokyphosis deformity that can benefit from AR, thereby optimizing 3D kyphosis restoration while eliminating unnecessary interventions.

## Methods

A prospective multicenter registry of AIS was used to identify patients undergoing PSF for main thoracic curves (Lenke 1–4) with at least 2 years of follow-up. This is a consecutive series of patients from sites that are allocated an annual enrollment allocation. Thoracic kyphosis measured by T5-T12 sagittal Cobb angle was converted to estimated 3D T5-T12 kyphosis by the previously validated formula: 3D kyphosis = 18.1 + (0.81*2D T5-T12 sagittal Cobb)—(0.54*2D coronal Cobb) [12]. Patients were included only if 3D kyphosis < 10° to limit to hypokyphotic patients. Additional patients were excluded if aged > 20 years at the time of surgery, coronal Cobb > 70°, or upper instrumented vertebra (UIV) caudal to T6 to isolate adolescent patients without extreme coronal deformities who had thoracic instrumentation.

Cohorts were defined by treatment with PSF only (PSF group) or the addition of anterior release (PSF + AR group) during the same surgical episode, though staged procedures separated by multiple days were also included. Univariate analysis of demographics, pre-operative characteristics, and surgical outcomes was performed between treatment groups with p-values generated from Pearson’s chi-square for categorical variables and Mann–Whitney for continuous variables. A subanalysis was performed which included only patients whose surgeons had contributed both PSF and PSF + AR patients to this study (surgeon ‘mutual’ to both groups), as an additional effort to control for the effect of the surgeon. Another subanalysis was performed, which only included surgeons whose mean kyphosis correction was above-average for the cohort (‘high-performing’: greater than 25.2°) to select those surgeons with presumed optimal technique and a surgical goal of kyphosis restoration. A linear mixed model was created to predict 2-year 3D kyphosis from the interaction of treatment by pre-op 3D kyphosis, while controlling for age, sex, thoracic coronal deformity and flexibility, number of posterior column osteotomies utilized, rod diameter, rod material, surgery recency, and surgeon. An additional model was created without an interaction term to assess the effect of the treatment approach, in general. A restricted cubic spline with 3 knots was included for surgery recency due to its nonlinear relationship with 2-year 3D kyphosis. Nonlinear relationships were checked for the other continuous predictors, including pre-op 3D kyphosis, but were not included in the final model due to a lack of significant nonlinear relationships. Predictor importance was ranked by the Wald *χ*^*2*^-d.f. [13] Missing values of the patient covariates were handled by multiple imputations. Statistical significance is defined by *p* < 0.05. Statistical analysis was performed by a biostatistician (JSP) using the *rms* package [14] in R version 4.3.0 (Vienna, Austria).

## Results

**Fig. 1:**
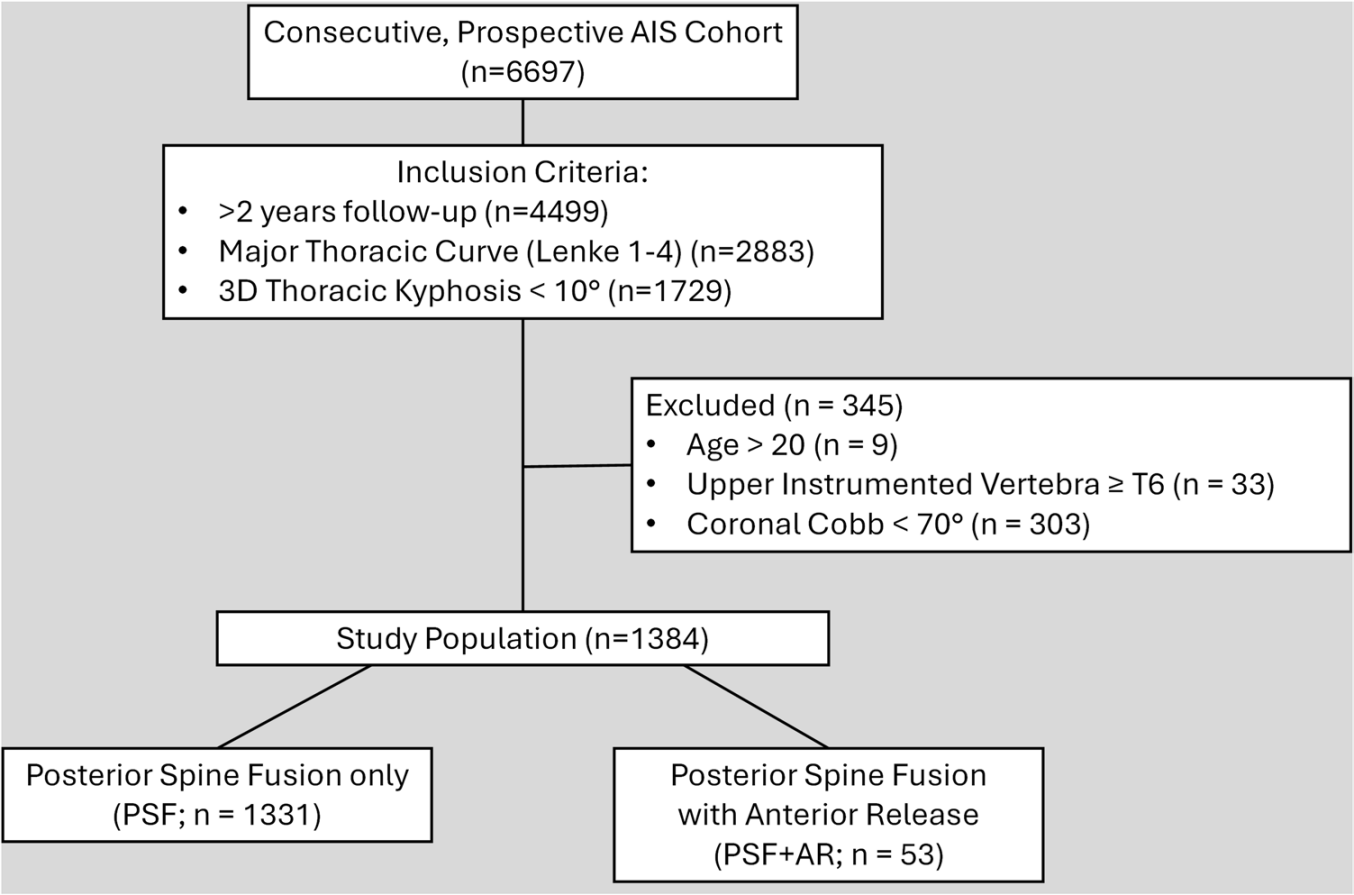
Flow chart demonstrating study-specific patient inclusion and exclusion from the overall study group AIS cohort

A total of 1384 patients met the inclusion and exclusion criteria (Fig. 1). Fifty-three (3.8%) had undergone PSF + AR, the remaining 1331 underwent isolated PSF. PSF + AR was performed in a staged fashion in 3 cases (6.4%) and anterior release was performed via thoracoscopy in 20 (37.7% of PSF + AR) cases. Table 1 demonstrates cohort demographics and pre-operative characteristics. PSF + AR patients were younger (13.9 vs. 14.8 years, *p* < 0.001) and had a higher frequency of open triradiate cartilage (36.5% vs. 7.2%, *p* < 0.001) compared to PSF. The majority of preoperative radiographic measurements were similar among groups, namely 3D kyphosis (− 3.7 ± 10.9° vs. − 0.5 ± 7.2°, *p* = 0.082) and coronal Cobb (54.6 ± 8.0° vs. 55.0 ± 7.2°, *p* = 0.674), though raw 2D kyphosis was less in the PSF + AR group (9.5 ± 12.6° vs. 13.7 ± 9.1°, *p* = 0.024). PSF + AR treatment was on average more recent.

**Table 1:** Cohort Characteristics

	PSF (*n* = 1331)	PSF + AR (*n* = 53)	*P*-value
Age at Surgery (years)	14.8 ± 2.0 years	13.9 ± 1.9 years	< 0.001^1^
Skeletal Immaturity (TRC open)	7.2%	36.5%	< 0.001^2^
% Male	19.5%	20.8%	0.828^2^
3D Thoracic Kyphosis (T5-T12) pre-operative	− 0.5 ± 7.2°	− 3.7 ± 10.9°	0.082^1^
2D Kyphosis (T5-T12) pre-operative	13.7 ± 9.1°	9.5 ± 12.6°	0.024^1^
Coronal Cobb (main thoracic)	55.0 ± 7.2°	54.6 ± 8.0°	0.674^1^
Coronal Bend (main thoracic)	34.6 ± 11.9°	33.4 ± 12.4°	0.649^1^
Lumbar Lordosis	− 56.0 ± 14.2°	− 53.6 ± 11.2°	0.183^1^
SRS-22 pre-operative	3.92 ± 0.5	3.94 ± 0.6	0.478^1^
Time since surgery	3310 ± 1679 days	1992 ± 2233 days	< 0.001^1^

x ± s represents mean ± 1 standard deviation for continuous variables. Tests used: Wilcoxon test; Pearson test

Surgical details varied among study cohorts (Table 2). Constructs were predominantly all pedicle screws with 5.5 mm diameter rods for both cohorts. Rod materials varied with stainless steel being most commonly used in the PSF group (49.8%) and cobalt chrome predominating in the PSF + AR group (72.0%; *p* < 0.001). The use of posterior column osteotomies (PCOs) was similar, with both number of PCOs per patient (4.16 ± 3.1 PSF vs. 4.57 ± 2.9 PSF + AR, *p* = 0.542) and the frequency of patients with PCOs (71.0% PSF vs. 83.0% PSF + AR, *p* = 0.057) being similar. Forty-eight surgeons were included with a variable distribution of patients in each study cohort.

**Table 2:** Surgical Characteristics

	PSF (*n* = 1331)	PSF + AR (*n* = 53)	*P*-value
Construct Type			0.071^2^
80% Screw	40.7% (535)	25.0% (13)	
All Screw	55.4% (728)	71.2% (37)	
Hybrid	3.9% (51)	3.8% (2)	
Largest Rod Diameter Used			0.396^2^
5.5 mm or less	90.9% (1199)	86.8% (46)	
6.0 mm	6.6% (87)	11.3% (6)	
6.35 mm	2.5% (33)	1.9% (1)	
Rod Material			< 0.001^2^
Cobalt Chrome	38.4% (502)	72.0% (36)	
Stainless Steel	49.8% (651)	12.0% (6)	
Titanium	8.3% (109)	10.0% (5)	
Hybrid	3.4% (45)	6.0% (3)	
Posterior Column Osteotomies (PCO)			
Patient received PCO	71.0% (945)	83.0% (44)	0.057^2^
Number of PCOs/patient	4.16 ± 3.1	4.57 ± 2.9	0.542^1^
Surgeon			< 0.001^2^

x ± s represents mean ± 1 standard deviation for continuous variables. Numbers after percentages represent frequencies. Tests used: ^1^Wilcoxon test; ^2^Pearson test

**Fig. 2:**
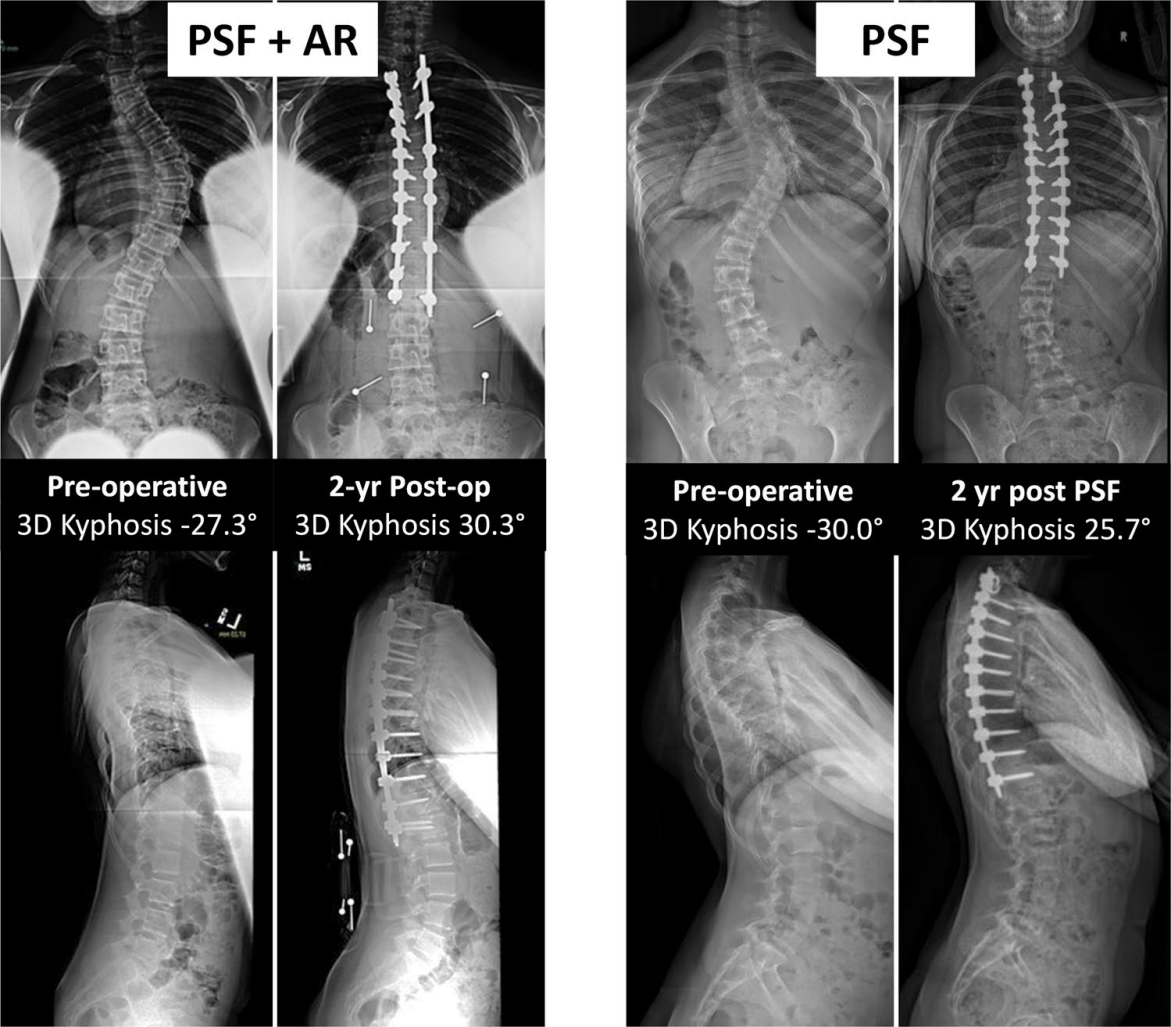
Pre- and post-operative radiographs of representative patients from each study cohort. Both cases demonstrate pre-operative thoracic lordosis, which was successfully corrected with Posterior Spine Fusion with Anterior Release (PSF + AR) or Posterior Spine Fusion alone (PSF)

Surgical correction appeared similar between PSF + AR and PSF in the unadjusted analysis (Table 3). 3D kyphosis showed dramatic improvement in both cohorts (26.7° ± 12.2° PSF + AR vs. 23.7 ± 8.6° PSF, p = 0.151) for comparable 2-year 3D kyphosis (23.3 ± 7.1° vs. 23.0 ± 9.1°, *p* = 0.925). There was no change in lumbar lordotic response between groups. The subanalyses for ‘mutual’ surgeons who contributed patients to both cohorts and for ‘high performing’ surgeons also did not demonstrate significant differences in 2-year estimated 3D kyphosis (Tables 4, 5). Representative cases are shown in Fig. 2. Documented blood loss and complications for the full cohort (Table 6) were similar among groups. PSF + AR had longer Operating Room times (323.7 vs. 266.3 min, *p* < 0.001) by nearly 1 h, yet showed decreased hospital length of stay (4.0 vs. 4.6 days, *p* = 0.003).

**Table 3:** Surgical and 2-Year Outcomes (Unadjusted)

	PSF (*n* = 1331)	PSF + AR (*n* = 53)	*P*-value
3D Thoracic Kyphosis at 2 years	23.3 ± 7.1°	23.0 ± 9.1°	0.925^1^
3D Thoracic Kyphosis Δ (pre to 2 yr)	23.7 ± 8.6°	26.7 ± 12.2°	0.151^1^
3D Thoracic Kyphosis > 20° at 2 yr (freq.)	66.4% (869)	71.7% (38)	0.422^2^
2D T5-T12 Kyphosis at 2 years	19.4 ± 7.6°	19.0 ± 9.3°	0.955^1^
2D T5-T12 Kyphosis Δ (pre to 2 yr)	5.7 ± 10.1°	9.5 ± 11.6°	0.019^1^
Lumbar lordosis response (pre—2 yr)	− 2.4 ± 14.1°	− 3.5 ± 19.5°	0.092^1^
Cumulative OR time	266.3 ± 90.8 min	323.7 ± 106.0 min	< 0.001^1^
Length of Hospitalization	4.6 ± 2.0 days	4.0 ± 2.3 days	0.003^1^
Blood Loss (mL)	815 ± 690 mL	913 ± 855 mL	0.743^1^
Blood Loss (% EBV)	0.235 ± 0.197	0.303 ± 0.288	0.179^1^
2-year post op SRS 22	4.4 ± 0.5	4.4 ± 0.4	0.777^1^

x ± s represents mean ± 1 standard deviation for continuous variables. Numbers after percentages represent frequencies. Tests used: ^1^Wilcoxon test; ^2^Pearson test. mL = milliliter, EBV = estimated blood volume

**Table 4:** ‘Mutual’ Surgeon Subanalysis (Unadjusted):

	PSF (does AR) (*n* = 654)	PSF + AR (*n* = 53)	*P*-value
Preoperative 3D Thoracic Kyphosis	− 0.6 ± 7.3°	−3.7 ± 10.9°	0.098^1^
3D Thoracic Kyphosis Δ (pre to 2 yr)	24.8 ± 8.9°	26.7 ± 12.2°	0.456^1^
3D Thoracic Kyphosis at 2 years	24.2 ± 6.7°	23.0 ± 9.1°	0.498^1^
3D Thoracic Kyphosis > 20° at 2 yr (freq.)	72.2% (466)	71.7% (38)	0.422^2^

Including Only ‘Mutual’ Surgeons with Patients in Each Cohort (excluding 677 PSF patients). x ± s represents mean ± 1 standard deviation for continuous variables. Numbers after percentages represent frequencies. Tests used: ^1^Wilcoxon test; ^2^Pearson test

**Table 5:** ‘High-Performing’ Subanalysis (Unadjusted):

	PSF (does AR) (*n* = 511)	PSF + AR (*n* = 30)	*P*-value
Preoperative 3D Thoracic Kyphosis	− 1.0 ± 7.5°	− 3.3 ± 11.1°	0.399^1^
3D Thoracic Kyphosis Δ (pre to 2 yr)	26.4 ± 8.6°	29.5 ± 11.9°	0.172^1^
3D Thoracic Kyphosis at 2 years	25.3 ± 6.5°	26.1 ± 7.3°	0.601^1^
3D Thoracic Kyphosis > 20° at 2 yr (freq.)	80.2% (404)	80.0% (24)	0.422^2^

Including Only “High-Performing” Surgeons with Patients in Each Cohort and > 25.2° Mean Kyphosis Restoration (9 surgeons). (excluding 820 PSF and 23 PSF + AR patients). x ± s represents mean ± 1 standard deviation for continuous variables. Numbers after percentages represent frequencies. Tests used: ^1^Wilcoxon test; ^2^Pearson test

**Table 6:** Complication Frequency (Unadjusted)

	PSF (*n* = 1331)	PSF + AR (*n* = 53)	*P*-value
Death	0	0	
Any Complication at 2 years (frequency)	12.4%	17.0%	0.324
Medical Complications	5.7%	9.4%	0.257
Gastrointestinal (GI)	2.0%	5.7%	0.065
Pulmonary	1.7%	1.9%	0.896
Other Medical	2.4%	3.8%	0.528
Surgical Complications	7.4%	9.4%	0.573
Neurologic Injury	3.5%	1.9%	0.536
Surgical Site Infection	3.0%	5.7%	0.275
Instrumentation Complication	1.1%	1.9%	0.612
Pseudoarthrosis	0	0	
Reoperations	2.2%	3.8%	0.442

Tests used: Pearson test

**Fig. 3:**
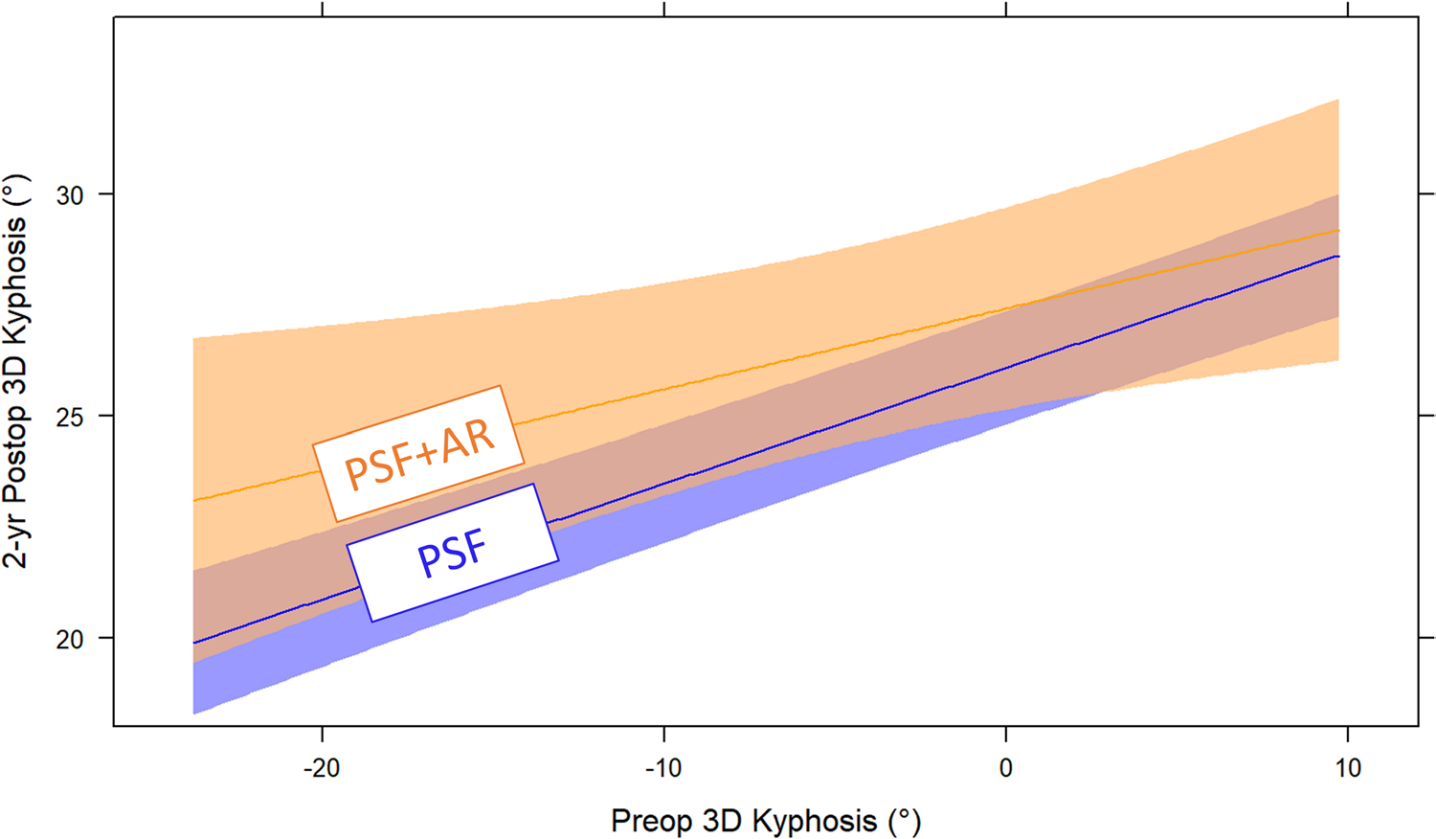
Linear mixed model predicting 2-year postoperative 3D kyphosis following PSF + AR vs. PSF depending on preoperative 3D kyphosis. Shaded area represents 95%CI. P = 0.31. The model explains 40% of variance in 2 yr 3D kyphosis outcome (R^2^ = 0.399)

**Fig. 4:**
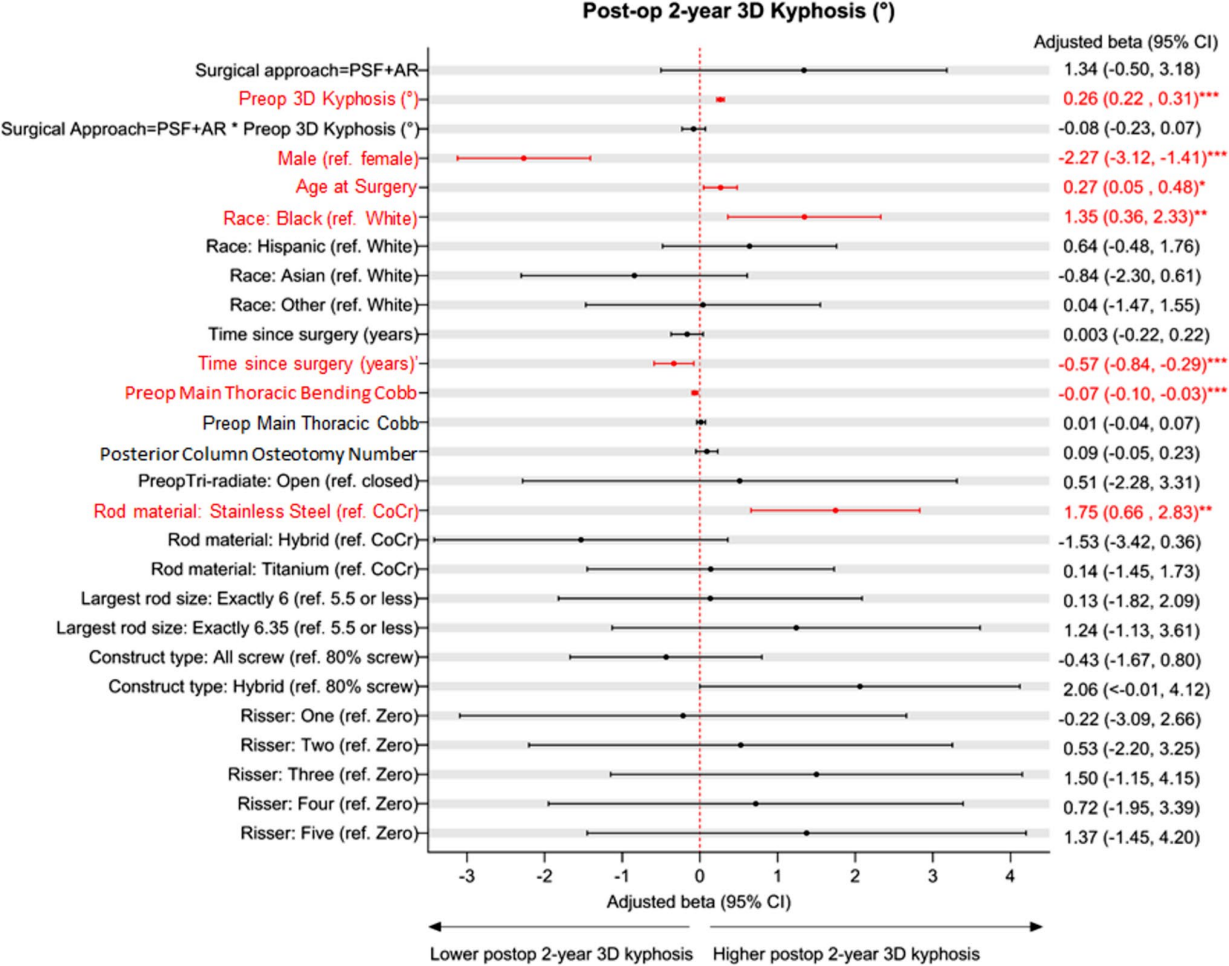
Forest plot for predictors of 2-year postoperative 3D kyphosis. Results shown are adjusted for surgeon, though individual surgeon effects not shown due to identification and space concerns. Error bars represent 95% CI. Red text indicates *p* < 0.05

**Fig. 5:**
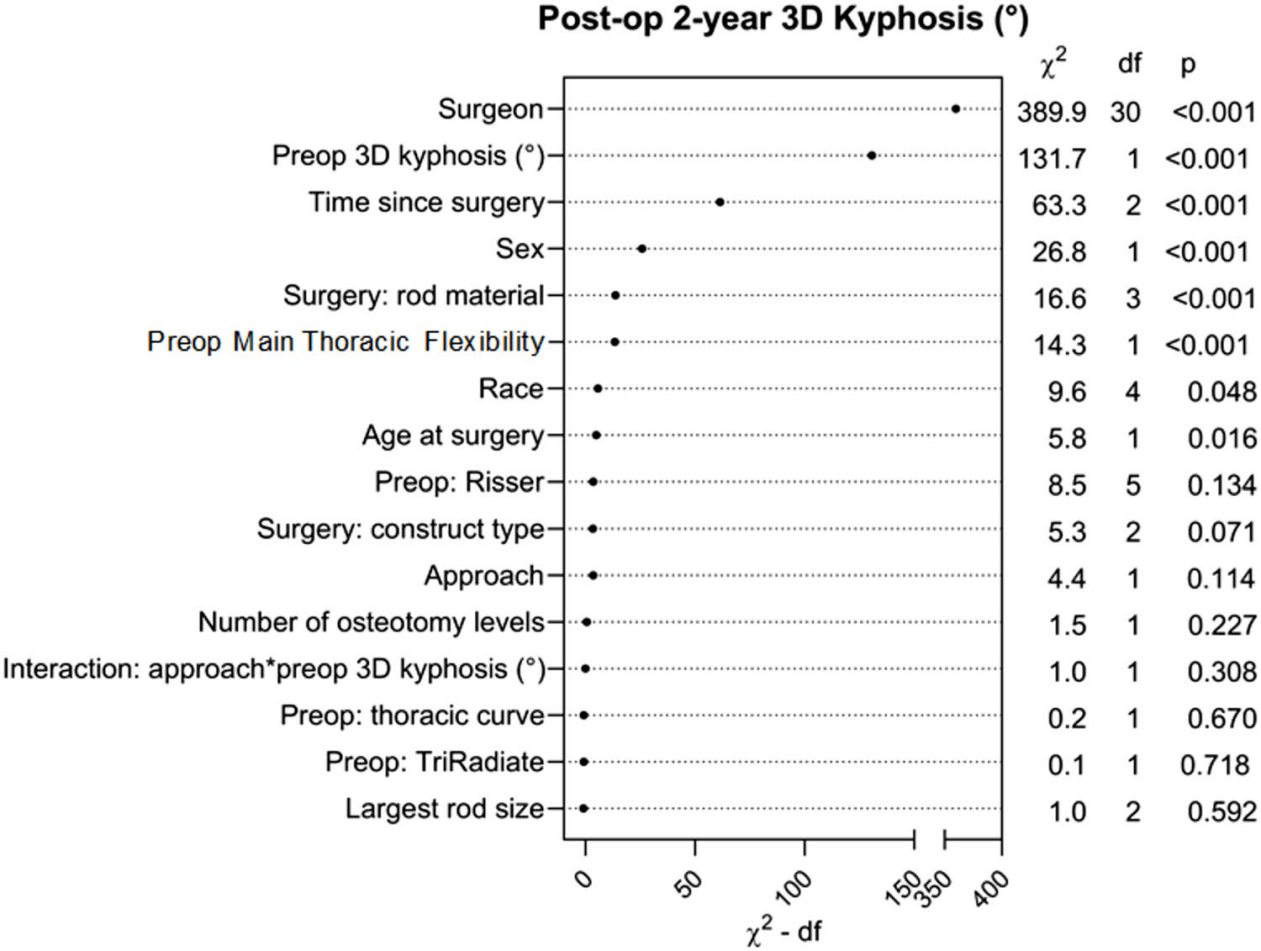
Predictor importance plot for prediction of 2-year postoperative 3D kyphosis

Linear mixed model for adjusted 3D kyphosis at 2 years post-op did not show an effect of the interaction of approach and preoperative 3D kyphosis (p = 0.31, adjusted β 1.34, 95% CI − 0.50–3.18; Fig. 3). Model for the treatment approach alone also did not show a statistical difference (*p* = 0.058, adjusted β 1.84, 95% CI − 0.06–3.75). Trendlines became more divergent at more extreme levels of preoperative 3D hypokyphosis deformity (< − 25°), but the sparse data in this range also do not support an effect of anterior release. The small difference in magnitude of kyphosis restoration between groups (about 3°) would be clinically insignificant, even if it were statistically significant. Post-hoc power analysis showed an adequate sample size to detect a difference of 5° in 2 yr 3D kyphosis between treatment approaches. Multiple other factors other than anterior release were associated with a significant increase in 2 yr 3D kyphosis, including surgeon identity, increased preoperative 3D kyphosis, female sex, older age, Black race, more recent surgery, smaller thoracic Cobb on flexibility films (more flexible), and use of stainless steel rod material. Figure 4 is the forest plot of model predictors. The predictor importance plot (Fig. 5) shows the relative importance of the surgeon compared to all other predictors. The model explains 40% of the variance in 2 yr 3D kyphosis outcome (*R*^*2*^ = 0.399).

## Discussion

Hypokyphosis or frank thoracic lordosis is a common component of AIS deformity, which can be difficult to correct. Historical series use anterior spine release in addition to PSF to improve sagittal correction, but it is unclear which degrees of deformity are severe enough to warrant this technique, if any. We hypothesized that PSF + AR would offer improved 2 yr 3D kyphosis over PSF alone for the most severe deformities. However, our linear mixed model did not reveal any significant effect of the treatment group or the interaction of treatment and preoperative 3D kyphosis on 2 yr 3D kyphosis outcomes when correcting for other predictors. The most significant predictor of 3D kyphosis restoration was surgeon identity, with patient and surgical factors playing lesser roles.

Prior investigations into the role of AR in modern spine surgery generally conclude that AR offers only modest benefit, if any, for coronal correction [9–11]. This is the first study, however, that evaluates the role of AR for hypokyphosis deformity not associated with a large coronal curve. Our results add further evidence that the benefits of AR for pure deformity correction are limited. There may still be a role for AR in the prevention of crankshaft phenomena for more skeletally immature patients, or perhaps larger lordotic deformities outside the range of this study population, though this study cannot address those specific scenarios.

Kyphosis restoration in AIS surgery has been the focus of multiple recent studies. Fletcher et. al. demonstrated that preoperative hypokyphosis deformity and smaller-diameter rods were risk factors for residual hypokyphosis in a single-center study [15]. Lonner et. al. used a multivariate model of 269 patients to evaluate factors associated with increased postoperative 2D kyphosis after AIS surgery, finding that an anterior approach for instrumentation and fusion was superior to PSF [16]. Only a small number of patients in that series had AR and PSF, so insight regarding AR effectiveness was limited. There is conflicting data regarding the effectiveness of modern posterior fusion and instrumentation techniques to restore kyphosis. Interestingly, the study by Bodendorfer et.al. lends insight to this phenomenon [17]. Their study indicates that kyphosis restoration in the era of early thoracic pedicle screws (2001–2009) was inferior to that of the prior era of primarily anterior instrumentation (1995–2000). Modern posterior instrumentation techniques (2010–2015) improved upon the early pedicle screw experience and approached that of the anterior techniques. This demonstrates that similar implants can yield vastly different results depending on the surgeons’ focus and the method in which implants are used. Work by Monazzam *et.al.* and Newton *et. al.* expand upon this point by implicating surgeon identity as the primary predictor of 2D and 3D kyphosis restoration, respectively, instead of the implant or surgical characteristics [18, 19].

The aforementioned studies were not designed to study the effect of AR and did not exclude large coronal deformities. This study utilized a large patient sample with linear mixed modeling focused specifically on the contributions of AR. While the present study did not demonstrate an important effect of AR on 2 yr 3D kyphosis, it confirms many of these prior findings, in particular the monumental effect that surgeon identity has on kyphosis restoration [18, 19]. We also observed a major effect of surgery recency in improving kyphosis restoration, like Bodendorfer et. al [17]*.* It is notable that these two factors played a much larger role in the model than any other technical factor, such as rod material. We attribute these findings to an increased focus on kyphosis restoration in modern treatment with varied adoption and execution among surgeons. Posterior column osteotomy number and rod size did not have any significant effect in this model. Patient-specific predictors, such as female sex and curve flexibility, have been previously noted to enable improved deformity correction and were confirmed in this study. It was surprising that the Black race and increasing age helped with kyphosis restoration in this study, as these are novel or conflicting findings. However, their importance as predictors is quite small and they contribute minimal variability to the model. Given the findings of the present study and consideration of past results, we think it is essential that both surgery recency and surgeon are included as covariates for future research into kyphosis restoration, and perhaps spine deformity research as a whole.

The decision to proceed with AR in addition to PSF is an impactful decision. AR is an additional surgical procedure done through a separate surgical approach, often in a distinct position. As such, AR necessitates increased surgical effort and time, and according to metanalyses, exposes a patient to additional risks of complications and blood loss compared to PSF alone [9]. In this study, however, anterior release did not lead to a statistically higher rate of complications. The similar rate of complications between groups can likely be attributed to the surgical skill and experience that the study group surgeons have with anterior spine surgery. As expected, with an additional surgical approach and procedure, the PSF + AR group did have increased blood loss and cumulative operating room time. Interestingly, the PSF + AR group had a shorter length of stay in unadjusted analysis, but this may be related to those surgeries being performed relatively more recently in the era of Enhanced Recovery pathways. Our study does support the notion that an AR can be performed in conjunction with PSF without significant added morbidity.

Limitations of the present study warrant discussion. While 3D kyphosis estimates are an accepted and validated measurement, they are not true 3D data from cross-sectional imaging. Using a 3D estimate afforded the ability to include more patients, which allowed us to obtain the necessary degrees of freedom to construct a linear mixed model inclusive of many variables. The ideal degree of thoracic kyphosis for an individual patient is likely variable, though currently understudied. Because patients in this study were all hypokyphotic at baseline, our study assumed the surgical goal was to obtain maximum kyphosis. We cannot know the surgeon’s goal for kyphosis restoration for any specific patient, and we cannot control for certain aspects of surgical technique (such as rod bending or reduction sequence). It is worth noting that our subanalysis investigating whether AR may make a difference for “high performing” surgeons with presumably “optimal technique” also showed no difference between cohorts. Additionally, the subanalysis investigating only surgeons with patients in each study cohort did not show a difference in kyphosis restoration for PSF and PSF + AR groups. While our cohort does represent the largest group of hypokyphotic AIS patients, those with severe lordosis (< − 40°) are underrepresented. This is primarily an AIS database, so all patients had coronal curves large enough to warrant surgery. It has been our observation that primary lordoscoliosis deformities, though rare, may be encountered where the coronal deformity is more modest, but there is a primary lordotic deformity. Our model cannot account for deformities outside of our sample range; therefore, these more severe deformities may yet demonstrate a benefit of anterior release.

In summary, these findings can inform treatment decisions for this population of moderate AIS with hypokyphosis deformities. Although kyphosis restoration can be challenging, it is an achievable goal with modern PSF techniques without the addition of AR. When AR is indicated for other deformities outside of the present study population, experienced surgeons can perform this without a significant increase in complications and only modest increases in operative time. Of all the factors in the model, surgeon identity and surgery recency have the largest effects on post-operative kyphosis, by a wide margin. These factors likely encompass evolving surgical characteristics that are difficult to quantify, such as the extent of soft tissue release, rod contouring, reduction maneuvers or sequence, or an overall prioritization of kyphosis restoration. Future research on corrective outcomes should attempt to isolate, control, and study these variables. At the very least, it is incumbent for future research on these topics to account for surgery recency and surgeon identity given that their influence is so great.

## Conclusion

In AIS patients with coronal curve < 70° and 3D hypokyphosis of 10° to − 40°, treatment with PSF + AR did not improve 2-year sagittal correction more than PSF alone. Surgeon identity and surgery recency influenced post-operative kyphosis more than any other patient or surgical factor.

## Data Availability

Aggregate Data is held in a protected database but is available to HSG surgeon members for study-related research projects.
